# Enhancing Conservation Efforts of *Stephanopodium engleri* Through Vegetative Propagation: Effects of IBA and Cutting Types

**DOI:** 10.3390/plants14142116

**Published:** 2025-07-09

**Authors:** Giselly Mota da Silva, Evandro Alves Vieira, Luiz Palhares Neto, Silvio Ramos, Markus Gastauer, Cecílio Frois Caldeira

**Affiliations:** 1 Vale Institute of Technology—ITV, Belém 66055-090, Brazil; giselly.silva@pq.itv.org (G.M.d.S.); evandro.vieira@pq.itv.org (E.A.V.); silvio.ramos@itv.org (S.R.); markus.gastauer@itv.org (M.G.); 2 Department of Biology, State University of Southwest Bahia, Jequié 45205-490, Brazil; netopalhares1@gmail.com

**Keywords:** branch cuttings, Dichapetalaceae, endangered woody plant species, plant conservation, Iron Quadrangle

## Abstract

*Stephanopodium engleri* Baill. is an endangered tree species from the Dichapetalaceae family and endemic to the Iron Quadrangle region of Brazil. Recalcitrance and low seed viability limit conventional seedling production, making vegetative propagation a crucial alternative for conservation efforts. This study evaluated the rooting and sprouting potential of different cutting types (apical, middle, and basal segments from the main stem, as well as the tip and the herbaceous and woody segments from the lateral branches) treated with Indole-3-Butyric Acid (IBA) at varying concentrations (0, 1, 2, 3, and 4 g L^−1^) and immersion durations (5 s to 10 min). Cuttings were collected from 12-month-old plants grown under controlled conditions and planted in Carolina Soil^®^ substrate after treatment. Sprouting and rooting rates varied significantly between cutting types, with basal main stem cuttings showing the highest rooting success, particularly at 3 g L^−1^ of IBA. These cuttings also exhibited more and longer roots and enhanced sprouting-related biometric traits. Shorter immersion times (15 s and 1 min) were the most effective, promoting root formation while avoiding the potential inhibitory effects of prolonged exposure. Our findings provide a practical protocol for large-scale seedling production of *S. engleri* while minimizing impacts on wild populations. The effective use of vegetative propagation could facilitate the expansion of *S. engleri* populations in their natural habitats, enhancing conservation efforts and ensuring sustainable species management.

## 1. Introduction

*Stephanopodium engleri* Baill. (Dichapetalaceae) is a tree species with occurrence restricted to the Semideciduous Seasonal Forest of Minas Gerais state, Brazil, and is considered a taxon of extremely rare occurrence [1,2]. The currently known populations of the species are limited to the remaining areas of the Atlantic Forest in the Iron Quadrangle and surrounding areas [3]. The Iron Quadrangle is a mineral-rich region located in the state of Minas Gerais (Brazil), known for its extensive deposits of iron ore, gold, and other minerals. It has undergone significant landscape changes due to mining activities [4] and the expansion of surrounding urban areas. A substantial habitat fragmentation driven by human activities resulted in diminishing the area of occurrence for *S. engleri* and exacerbating the risk of extinction for the species [2]. In fact, due to the absence of herbarium records for over 50 years, *S. engleri* was then classified as “Probably Extinct” in 1997 [3]. However, subsequent findings in the 2000s prompted a reevaluation, leading to its reclassification as an endangered species according to the IUCN criteria [1]. These recent records enable the implementation of conservation initiatives geared toward mitigating the risk of extinction.

Effective conservation strategies for endangered species such as *S. engleri* require not only the protection and enhancement of the connectivity of the remaining areas but also activities aimed at strengthening the existing populations and/or reintroducing individuals into suitable areas to establish a viable and self-sustaining population [5]. Adding new individuals to populations can increase genetic diversity and also positively influence the dynamics of mating opportunities and the potential for pollinator visits. These interventions are crucial for minimizing potential genetic drifts and fortifying the species’ adaptive capacity in response to habitat alterations and changing climatic scenarios [6,7]. Considering that propagation knowledge is fundamental to obtaining new individuals, it represents one of the first steps towards the conservation of a threatened species by allowing proficient seedling production [8]. Despite its ecological and conservation significance, research into the propagation of *S. engleri* is limited. This is largely due to the limited availability of mature individuals capable of producing viable seeds. Furthermore, factors such as seed predation and the extensive efforts required to locate existing specimens or conduct propagation studies hinder research progress.

While seed-based seedling production is desirable due to advantages such as the potential to enhance genetic diversity, ease of execution, and lower seedling production costs, the species *S. engleri* faces limitations in obtaining seedlings through this method. In addition to the limited number of known reproductive adult plants, recent studies indicate that seeds of this species are recalcitrant, i.e., they rapidly lose viability after fruit maturation and dispersal, posing challenges in the storage and maintenance of seed viability [9]. Nevertheless, given the vulnerability status of *S. engleri* and the imperative need for seedlings to support conservation efforts, vegetative propagation emerges as a promising alternative to augment seedling multiplication. Seedlings obtained from vegetative propagation hold the potential for enhancing natural population diversity and studies on habitat suitability in the search for areas to establish new populations.

Vegetative propagation is a widely used method for the large-scale production of economically important plants in the horticultural, forestry, and agricultural sectors [10]. Propagation by cuttings is particularly notable among these methods for its simplicity, low cost, and compatibility with standard nursery infrastructure, making it especially suitable for replicating tree species such as *S. engleri*. Adventitious root and shoot developments in cuttings are the necessary steps for effective vegetative propagation. The main factors that regulate this process are (1) internal factors, including plant age and health, plant organs, vigor and plant growth regulators (PGR, especially auxins), carbohydrates, and other compounds, and (2) external factors, such as temperature, light, growing medium, mineral source, application of exogenous hormones, and immersion time of the cuttings into the exogenous hormones [11,12,13]. The position of the cuttings in the plant has a great influence on rooting, both in terms of phytohormonal issues and the age of the tissues. Apical cuttings are younger and have greater auxin synthesis but dehydrate more easily [14,15]. Basal cuttings, which are more lignified and have a low endogenous auxin content, have greater reserve storage, which may be advantageous for the emergence and growth of shoots and roots [15].

Successful vegetative propagation via cuttings usually requires the exogenous application of plant regulators, especially auxins. Auxins are directly involved in cell division and expansion, or indirectly interact with other phytohormones or molecules in the base of cuttings, favoring the formation of adventitious rooting. Likewise, auxins also influence the early stages of embryogenesis, apical meristem organization, and the branching aerial parts [10]. The formation of adventitious roots includes the formation of meristematic cells niches, as initial cells and/or target cells [16]. In general, treated cuttings with the auxin indole butyric acid (IBA) show high rooting and sprouting [17,18], although high concentrations of IBA can also hinder the rooting process due to phytotoxicity in some species [11,19].

Immersion of the proximal ends of cuttings in an IBA solution promotes adventitious rooting and sprouting in tree species across various families, such as *Castanea sativa* [20], *Argania spinosa* [21], *Gliricidia sepium* [22], *Syzygium samarangense* [23], and *Morus alba* [24]. Due to challenges in seedling production from seeds and a dearth of knowledge regarding the vegetative propagation of the *Stephanopodium* genus, this study aimed to investigate the potential for seedling multiplication through cuttings taken from various plant portions (branches and main stem). We also examined the effect of varying concentrations and immersion durations of the plant growth regulator IBA. Our hypothesis posits that rooting and sprouting in *S. engleri* will vary based on (i) the source of cuttings from different plant parts and (ii) the immersion duration and concentration of the IBA solution.

## 2. Materials and Methods

### 2.1. Plant Material

The study was carried out in the Plant Growth Laboratory of the Vale Institute of Technology, Belém, Brazil. Seeds of *S. engleri* harvested from adult plants in forest fragments near Belo Horizonte (Brazil) were used to obtain seedlings. Access to the plants at these sites was kindly granted by Vale S.A. and Gerdau S.A. Voucher specimens from these plants were previously deposited in the BHZB (no. 11303) and CVRD (no. 15967) herbaria. Under greenhouse conditions, 40 seedlings were grown for 12 months. Daily air temperatures (measured with RHT10, Extech Instruments, Boston, MA, USA) ranged from 25 to 37 °C, while the vapor pressure deficit (VPD) fluctuated between 0.4 and 2.5 kPa. The photoperiod remained relatively stable throughout the year at approximately 12 h of daylight and 12 h of darkness, consistent with the site’s equatorial location (latitude ~1.45° S). The midday photosynthetic photon flux density (PPFD) was periodically measured using a quantum sensor (LI-190R, LI-COR, Lincoln, NE, USA) and varied between 900 and 1500 µmol m^−2^ s^−1^. When the plants reached a 1.5 cm shoot diameter and 50–60 cm height, they were selected as donors to provide the cuttings used in this study. The main branches were detached from the mother plants, temporarily placed in a polystyrene container with water, and covered with a transparent plastic film to avoid dehydration and tissue oxidation.

#### Cutting Preparation and Growth Conditions

Uniform-sized cuttings were prepared and treated according to the specified protocols before being transferred to a growth chamber with controlled environmental conditions. Following the treatments described below, all cuttings were maintained in a high-humidity environment near the dew point by covering the trays with a transparent plastic sheet until the final evaluation. The photoperiod was set to 12 h of light and 12 h of darkness, with a light intensity of 70 µmol m^−2^ s^−1^. Day and night temperatures were maintained at 28 °C and 22 °C, respectively. The cuttings were watered daily using a manual sprinkler inside the covered environment. This procedure was consistently applied across all subsequent experiments, encompassing both the main stem and lateral branch cuttings.

### 2.2. Propagation with Main-Branch Cuttings

#### 2.2.1. Different Concentrations of IBA

The main branches were divided into three segments to obtain the different types of cuttings: apical, middle, and basal, whose average diameters were 4, 6, and 8 mm, respectively. The cuttings were standardized with at least two buds, approximately 10 cm in length, and all of the leaves were removed. The cuttings were extracted continuously along the length of the branch. For rooting, the base of the cutting (1.0 cm) was immersed for 5 s in an IBA solution containing 0 (control), 1, 2, 3, and 4 g L^−1^. The cuttings were planted in trays with cells of 200 mL filled with Caroline Soil^®^ (a commercial organic-rich substrate) and transferred to a growth chamber with controlled environmental conditions. A completely randomized design was used, with fifteen treatments (3 types of cuttings × 5 doses of IBA) and eight replications, for a total of 120 cuttings. At 45 days after planting, the following parameters were evaluated: sprouting rate (%, number of cuttings sprouting/number of cutting in each replicate), number of shoots, rooting rate (%, number of cuttings rooting/number of cutting in each replicate), number of roots, and main root length (cm).

#### 2.2.2. Immersion Durations

In this essay, cuttings from the middle and apical parts were combined into a single group (middle + apical) due to the uniformity of the diameters (0.5 cm), while the basal samples had a thickness of about 0.7 cm. The IBA concentration used was 3 g L^−1^ (established from previous trials), with three different immersion times: 15 sec and 1 and 2 min. All environmental conditions were the same as in the previous work. The experiment was conducted using a completely randomized design, comprising six treatments arranged in a 2 × 3 factorial scheme (two types of cuttings × three soaking durations). Each treatment included ten replications for basal cuttings and seven replications for middle and apical cuttings. Also, the same variables were evaluated after 45 days of planting, including the height of shoots.

### 2.3. Propagation with Lateral Branch-Cuttings

#### 2.3.1. Different Concentrations of IBA

Tip cuttings and woody and herbaceous lateral branches, with average diameters of 3.5, 2.3, and 2 mm, respectively, were evaluated for shoot development and rooting. The standardization used for the cuttings was the presence of two buds and 10 cm in length. In an effort to balance water loss with photosynthetic capacity, a pair of halved leaves was retained on both tip cuttings and woody branches, whereas a pair of whole young leaves was maintained on herbaceous cuttings. For rooting, rapid immersion (5 s) was used with two concentrations of IBA: 0 (control) and 2 g L^−1^. After immersion, the cuttings were planted in the Carolina Soil^®^ substrate. The cuttings were also kept in distilled water to verify rooting in the absence of a growth regulator. The cuttings were kept under the same growth conditions as in the previous experiments. The experimental design was completely randomized, with nine treatments (3 types of cuttings × 3 rooting conditions). The number of replicates was 16 cuttings per treatment. At 45 days after planting, the following parameters were evaluated: sprouting rate (%, number of cuttings sprouting/number of cutting in each replicate), number of shoots, rooting rate (%, number of cuttings rooting/number of cutting in each replicate), number of roots, and main root length (cm).

#### 2.3.2. Immersion Durations

In this experiment, lateral cuttings were immersed in a solution with an IBA concentration of 3 g L^−1^ for five durations: 15 s, 1, 2, 5, and 10 min. The cultivation method and the commercial substrate used were the same as in the previous test. Also, the same variables were evaluated after 45 days of planting. The experimental design was completely randomized with five treatments and 25 replications each.

### 2.4. Data Analysis

The data were tested for normality using the Shapiro–Wilk test (*p* > 0.05) and for homogeneity of variances using Bartlett’s test (*p* > 0.05). Subsequently, a one-way analysis of variance (ANOVA) was performed. For the sprouting and rooting rates, significance was assessed using Cochran’s Q test at a 5% significance level (*p* < 0.05). For other variables showing significant differences, means were compared using Tukey’s test at a 5% probability level. All statistical analyses were conducted in the R environment using the RStudio v4.2.1 interface [25].

## 3. Results

### 3.1. Propagation with Main Stem

#### 3.1.1. Cutting Positions and Concentrations of IBA

**Figure 1:**
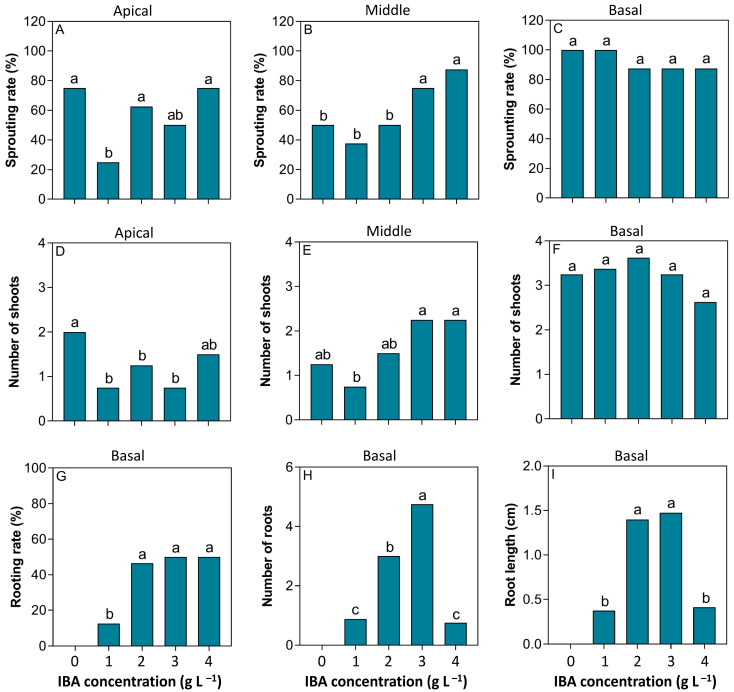
The effect of increasing IBA concentrations on sprouting and rooting of *Stephanopodium engleri* main stem cutting types. Sprouting rate of apical (**A**), middle (**B**), and basal (**C**) cuttings; Number of shoots for the apical (**D**), middle (**E**), and base (**F**) cuttings; rooting rate (**G**), number of roots (**H**) and root length (**I**) from base cuttings. Different letters in each box indicate significant differences between means by the Cochran’s test (*p* < 0.05) for sprouting rate and rooting rate. The other variables with significant differences had their means compared by the Tukey test (*p* < 0.05).

**Figure 2:**
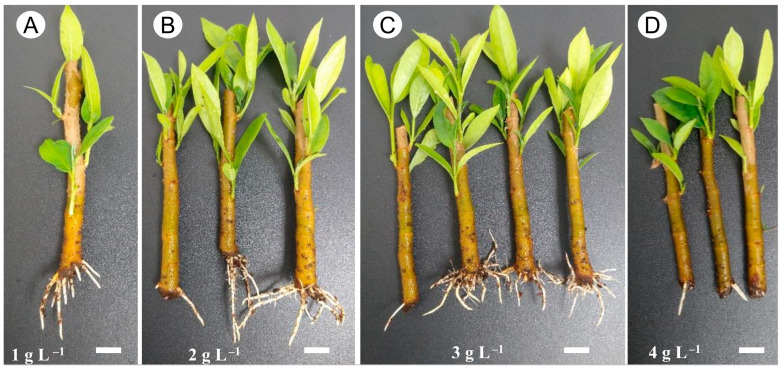
Images of basal cuttings of the main stem of *Stephanopodium engleri* rooting at different IBA concentrations: (**A**) 1 g L^−1^, (**B**) 2 g L^−1^, (**C**) 3 g L^−1^, and (**D**) 4 g L^−1^. Cuttings in the control treatment are not shown because they did not root and dried before the end of the experimental period. (Bar: 1 cm).

The basal cuttings exhibited a sprouting rate greater than 85% for all IBA treatments, with no significant differences observed between treatments, and complete sprouting (100%) was achieved with 0 and 1 g L^−1^ of IBA. The middle cuttings showed higher sprouting rates when treated with 3 and 4 g L^−1^, reaching almost 80%. Conversely, apical cuttings showed lower sprouting rates, with maximum sprouting observed in all treatments except those treated with 1 g L^−1^ (Figure 1 and Figure 2A–C). Overall, the basal cuttings developed a greater number of branches, with no significant differences observed between treatments. For the middle cuttings, branching was positively correlated with higher IBA concentrations, while no clear trend was observed for the apical cuttings (Figure 1D–F).

Rooting was observed only in the basal cuttings treated with doses above 1 g L^−1^ of IBA (Figure 1 and Figure 2). In addition, a higher number of roots and greater root length were observed, particularly in the basal cuttings treated with 2 and 3 g L^−1^ of IBA, all of which showed over 50% rooting success (Figure 1G–I).

#### 3.1.2. Immersion Times

**Figure 3:**
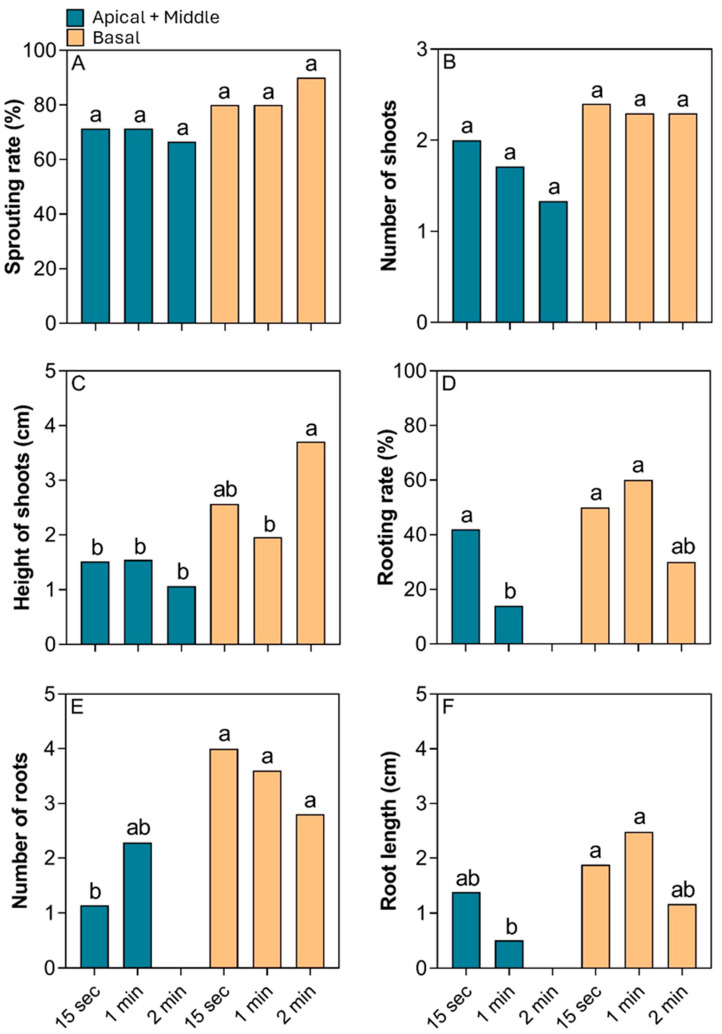
Effect of different IBA soaking times and types of cuttings of the main branch (basal and middle + apical) on the (**A**) sprouting rate, (**B**) number of shoots, (**C**) height of shoots, (**D**) rooting rate, (**E**) number of roots, and (**F**) root length in *Stephanopodium engleri*. Different letters in each box indicate significant differences between means by the Cochran’s test (*p* < 0.05) for sprouting rate and rooting rate. The other variables with significant differences had their means compared by the Tukey test (*p* < 0.05).

**Figure 4:**
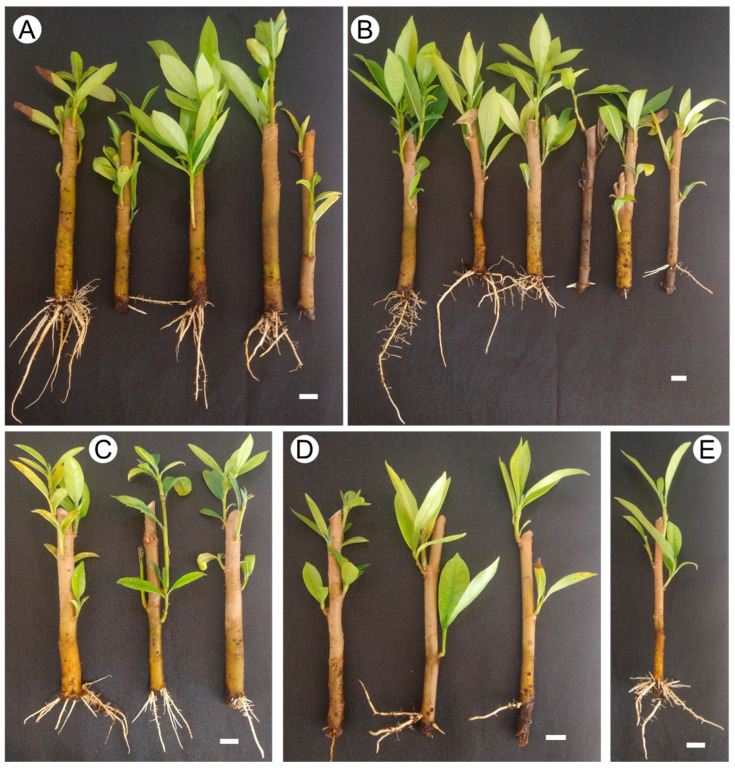
Sprouting and rooting in cuttings of the main stem in *Stephanopodium engleri* subjected to different soaking times in 3 g L^−1^ of IBA. (**A**–**C**) basal cuttings; (**A**) 15 s; (**B**) 1 min; (**C**) 2 min; (**D**,**E**) middle and apical cuttings; (**D**) 15 s; (**E**) 1 min. (Bar: 1 cm).

The basal cuttings showed the highest sprouting rate and number of shoots, although no significant differences were observed (Figure 3A,B). However, the basal cuttings immersed in IBA for 15 s and 1 min showed greater shoot height compared to apical + middle cuttings, which showed less growth (Figure 3C). For the root system, the basal cuttings immersed in IBA for 15 s and 1 min showed higher rooting, with no more than 50% of the cuttings successfully rooting. Similarly, the apical + middle cuttings exhibited higher rooting (43%) with 15 s of immersion, while longer immersion times (1 and 2 min) resulted in reduced rooting success, with 14% and complete inhibition, respectively (Figure 3D). A similar pattern was observed for the number of roots and root length for both types of cuttings (Figure 3E,F). As shown in Figure 4A–C, basal produced better results with more branched roots and shoots with more leaves.

### 3.2. Propagation with Branch Cuttings

#### 3.2.1. Different Concentrations of IBA

**Figure 5:**
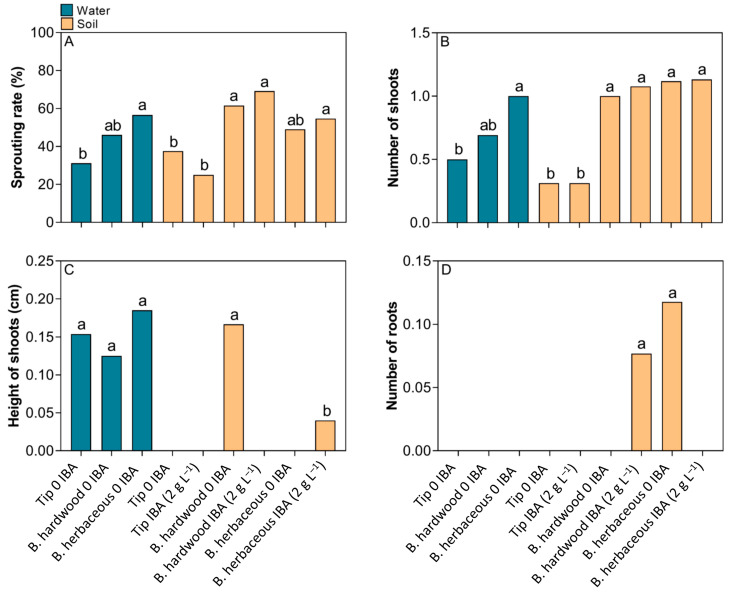
Sprouting and rooting of tip and branch cuttings (hardwood and herbaceous) of *Stephanopodium engleri* immersed in different IBA concentrations and transferred to water or soil substrate. (**A**) Sprouting rate, (**B**) number of shoots, (**C**) height of shoots, and (**D**) number of roots. Different letters in each box indicate significant differences between means by the Cochran’s test (*p* < 0.05) for sprouting rate. The other variables with significant differences had their means compared by the Tukey test (*p* < 0.05).

The highest sprouting rate (69%) and number of shoots were observed in cuttings from woody branches treated with 2 g L^−1^ of IBA, followed by herbaceous cuttings, regardless of whether they were grown in water or soil (Figure 5A,B). Conversely, the lowest sprouting rates (25%) and the lowest number of shoots were observed in tip cuttings immersed in IBA, especially in the control and 2 g L^−1^ of IBA in soil (Figure 5A,B). For the shoot, herbaceous cuttings in water (0 IBA) and hardwood (0 IBA) in soil showed greater shoot height, while the opposite effect was observed for herbaceous cuttings with 2 g L^−1^ of IBA in the soil. In addition, some treatments showed no increase in shoot height (Figure 5C). Rooting was absent in the majority of treatments. However, the highest number of roots was observed in the herbaceous cuttings without IBA application and in the hardwood cuttings treated with 2 g L^−1^ of IBA, with both cultivated in soil (Figure 5D).

#### 3.2.2. Immersion Times

**Figure 6:**
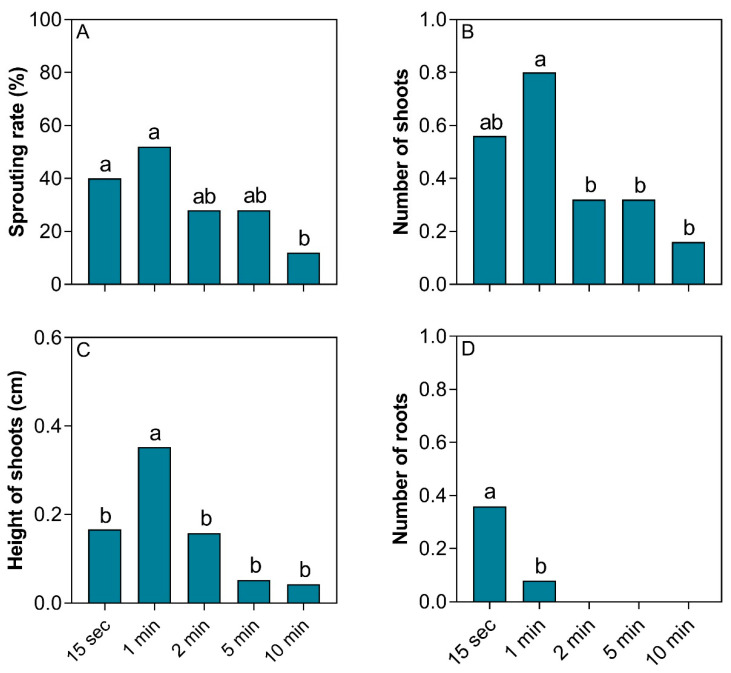
The effect of immersion time on sprouting and rooting of *Stephanopodium engleri* branch cutting. (**A**) Sprouting rate, (**B**) number of shoots, (**C**) height of shoots, and (**D**) number of roots. Different letters in each box indicate significant differences between means by the Cochran’s test (*p* < 0.05) for sprouting rate. The other variables with significant differences had their means compared by the Tukey test (*p* < 0.05).

Immersion of the cuttings for 15 sec and 1 min significantly increased the sprouting rate and the number of shoots (Figure 6A,B). Conversely, only immersion for 1 min resulted in an increased shoot height, which was significantly different from the other treatments (Figure 6C). Regarding rooting, only short periods of immersion (maximum 1 min) were able to induce root emissions. In fact, the shorter the time (15 s), the higher the number of roots observed (Figure 6D).

## 4. Discussion

### 4.1. Vegetative Propagation Can Be an Effective Approach for S. engleri Seedling Production

While vegetative propagation is widely utilized for large-scale plant production, its success depends on numerous factors, often making it a challenging endeavor. Like the hurdles faced with *S. engleri*, many studies report difficulties in establishing efficient propagation protocols, particularly regarding rooting. For example, [26] reported excellent survival and sprouting rates in *Sambucus australis*, but low rooting percentages limited propagation success. In *Escallonia bifida*, excessive moisture led to the complete failure of cuttings due to rotting. Similarly, *Bertholletia excelsa* cuttings showed variable rooting percentages (8–58%) depending on cutting position and IBA concentration [27], reflecting a similar variability seen in *S. engleri*. These findings underscore the complexity of vegetative propagation, where species-specific traits are critical to rooting success. This was further emphasized by [28] in a study of 20 forest species, where rooting rates varied widely (0.5–88%), influenced by factors such as cutting diameter. Consistently, cuttings with larger diameters yielded better seedling traits, a trend also observed in *S. engleri*.

In this study, we observed that basal cuttings of the main stem exhibited significantly higher root and shoot growth compared to middle and apical cuttings, which demonstrated minimal rooting and produced fewer healthy plants. This trend is consistent with findings in other species, where basal cuttings tend to show superior rooting efficiency [29,30]. Cutting sizes (length and diameter) are usually determinant of seedlings’ survival and growth in normal propagation conditions [31]. The enhanced performance of basal cuttings can be attributed greater capacity to provide reserves and to the concentration of endogenous root-promoting substances [32]. Additionally, basal cuttings, being larger, often have more buds and, consequently, a higher sprouting rate to rebuild the shoot organs. Together with more nutrient reserves, it would facilitate a higher nutrient translocation rate towards the rooting zone and supporting root formation [33,34].

The IBA application on *S. engleri* cuttings from the main stem, especially 2–3 g L^−1^ of IBA, further improved rooting and sprouting, likely by stimulating cambium activity and enhancing carbohydrate mobilization [35]. Optimal IBA concentrations can promote starch breakdown and accelerate carbohydrate movement from leaves to cuttings, providing the energy and carbon skeletons required for new tissue development [36,37]. The larger number of new leaves coming from the high sprouting rate from the basal cuttings in *S. engleri* may have played a fundamental role, acting as a carbohydrate source, as observed for other species [38].

Regarding immersion time, shorter durations (15 s and 1 min) were most effective for rooting. This immersion time can vary with species-specific requirements, as some plants benefit from prolonged immersion, while others respond better to brief exposure. For example, *Salix nigra* showed increased biomass with a 10-day immersion period [39], whereas *Conocarpus erectus* achieved maximum rooting with a 1 h IBA treatment [40]. Conversely, *B. excelsa* cuttings rooted best with only 1 s of IBA immersion at 1 g L^−1^ [27]. In *S. engleri*, prolonged immersion (2 min) inhibited root development in basal cuttings and completely suppressed rooting in middle and apical cuttings. However, a 2 min immersion slightly improved shoot height in the basal cuttings. This indicates that shorter immersion times are generally more effective for rooting and sprouting, accelerating the propagation process for large-scale seedling production.

### 4.2. Despite Vigorous Shoot Sprouting in Overall Cutting Types, the Rooting Rate Remained Low Even After Auxin Treatments

Our results evidenced that *S. engleri* cuttings exhibited high sprouting rates across almost all types. However, the rooting success remained limited, even with IBA treatments. Such a disconnect between shoot and root development is common in vegetative propagation and highlights the challenges associated with synchronizing these processes. Similar findings were reported for *Acalypha wilkesiana*, where hard–woody and semi-woody cuttings outperformed herbaceous ones due to greater lignification and nutrient reserves [41]. While the smaller-diameter cuttings of *S. engleri* demonstrated robust sprouting, their inability to establish a functional root system capable of supporting water and nutrient uptake limited their potential for successful propagation. As the more herbaceous cuttings dispose of fewer nutrient reserves, they depend on active photosynthesis to sustain rooting processes, making them more susceptible to environmental limitations [42,43]. The application of IBA, varying in concentration and immersion times, may often solve this bottleneck and enhance rooting [44], but we yielded mixed results within this study. Future optimization of vegetative propagation protocols for *S. engleri* should focus on refining cutting selection and auxin treatments to improve rooting rates, especially for the more abundant cutting types, such as those with smaller diameters.

Cutting type and auxin treatment significantly influenced the vegetative propagation success of *S. engleri*. Hard–woody cuttings treated with 2 g L^−1^ of IBA in soil exhibited higher sprouting rates and shoot numbers compared to herbaceous and tip cuttings, highlighting the role of lignification and nutrient reserves in propagation success, as also observed in *Acalypha wilkesiana* and *Bougainvillea buttiana* [41,45]. The presence of multiple viable meristematic zones in *S. engleri* may have further enhanced sprouting, particularly in soil-grown cuttings, aligning with [44], who suggested that shoot meristems and young leaves in softwood cuttings sustain endogenous auxin supply.

Despite vigorous sprouting, rooting remained limited across most treatments, emphasizing the importance of selecting the appropriate cutting type for successful propagation. Hardwood cuttings treated with 2 g L^−1^ of IBA and herbaceous cuttings without IBA in soil exhibited the highest root production, while other treatments showed poor or no rooting. This may be attributed to carbohydrate storage differences, as hardwood cuttings typically contain higher carbohydrate reserves, facilitating root formation [46]. Additionally, leafy cuttings may benefit from current photosynthate production, whereas hardwood cuttings rely on stored reserves [42,43]. Similar trends were observed in *Bertholletia excelsa*, where semi-hardwood cuttings treated with IBA exhibited superior rooting compared to untreated cuttings [47]. These findings underscore the complexity of vegetative propagation in *S. engleri* and the need for species-specific protocols to optimize rooting success.

### 4.3. Auxin Sensitivity Varies with Cutting Age and Position in Stephanopodium engleri Propagation

Our findings indicate that cutting position and IBA concentration significantly influence the vegetative propagation success of *S. engleri*. Regardless of the treatment, hardwood and herbaceous branch cuttings exhibited higher sprouting rates and a greater number of shoots compared to lateral and tip cuttings. These cuttings also developed the most roots, whereas tip cuttings failed to produce any. The variation in rooting ability between young lateral or tip cuttings and other branch sections may be attributed to differences in carbohydrate availability and/or endogenous auxin distribution along the shoot axis. The balance between carbohydrates and hormones plays a crucial role in determining whether cuttings prioritize sprouting or rooting. As suggested by [48], increased disaccharide and starch utilization could shift carbohydrate allocation towards root formation instead of promoting shoot growth. In *S. engleri*, young cuttings treated with 2000 mg L^−1^ IBA optimized sprouting at the expense of rooting, indicating that juvenility may affect the hormone response.

Similar trends were observed in the branch cuttings submerged in 3 g L^−1^ IBA for 15 sec and 1 min, which promoted higher sprouting rates, increased shoot numbers, and greater shoot height. However, prolonged immersion times at this concentration led to reduced rooting and root numbers, suggesting a potential inhibitory or toxic effect of IBA. High auxin concentrations or extended exposure times can act as root growth inhibitors, as reported in other species [11,31,49]. Ficko & Naeth (2022) [50] further highlighted that species-specific auxin thresholds exist, beyond which excessive hormone levels negatively impact rooting success. In *S. engleri*, the observed inhibition at 3 g L^−1^ IBA suggests that optimizing the hormone concentration and the exposure time is critical for balancing shoot and root development in vegetative propagation protocols.

## 5. Conclusions

Vegetative propagation of *Stephanopodium engleri* offers a practical and effective alternative for seedling production, addressing the species’ limited seed availability and short post-harvest viability. The superior rooting and sprouting performance observed in basal cuttings treated with 3 g L^−1^ of IBA for short immersion durations (15 s to 1 min) highlights a promising technique for large-scale propagation that minimizes the impact on natural populations. These findings contribute to the development of propagation strategies that support both experimental applications and conservation initiatives. Future research should aim to refine these protocols by investigating the physiological and environmental factors influencing propagation success, evaluating the long-term performance of propagated individuals in restoration contexts, and exploring complementary ex situ methods such as plant tissue culture to enhance genetic conservation and propagation efficiency. Such efforts will be essential for informing conservation planning and ensuring the sustainable management of this rare and endangered species.

## Data Availability

The data that support the findings of this study are available from the corresponding author upon reasonable request.
